# Genotyping *Echinococcus granulosus* from Canine Isolates in Ilam Province, West of Iran

**Published:** 2017

**Authors:** Abdolhossein DALIMI, Morteza SHAMSI, Afra KHOSRAVI, Fatemeh GHAFFARIFAR

**Affiliations:** 1. Dept. of Parasitology, Faculty of Medical Sciences, Tarbiat Modares University, Tehran, Iran; 2. Dept. of Immunology, School of Medicine, Ilam University of Medical Sciences, Ilam, Iran

**Keywords:** *Echinococcus granulosus*, Stray dog, Golden jackal, Genotyping, Iran

## Abstract

**Background:**

Cystic Echinococcosis (CE) is one of the most common parasitic zoonosis caused by *Echinococcus granulosus* worldwide. This study investigated genotype diversity of *Echinococcus granulosus* isolated from stray dogs and golden jackals in Ilam province, West of Iran.

**Methods:**

Adult worms were collected from the small intestine of the stray dogs and golden jackals from Ilam Province roads during 2012-2014. DNA was extracted from the adult worms and the partial mitochondrial NADH dehydrogenase subunit1 (*nad1*) was amplified by PCR then the products were digested by using HpaII*, Rsa*1, and *Alu*1 restriction enzymes. In order to confirm RFLP results, a number of PCR products were bi-directionally sequenced.

**Results:**

Totally, 20 stray dogs out of 75 (26.66%) and two out of 73 (2.74%) golden jackal showed infection with *E. granulosus*. Amplified PCR product for all isolates was a band of approximately 550bp. *Alu*1 digested the product into two bands of approximately 160bp and 390bp fragments, while the *Rsa1* cut the product into 320bp and 230bp fragments and the *Hpa*II had no effect on the PCR product for both dog and jackal samples. The isolate sequences of mtDNA*nad1* gene indicated 100% homology with references G1, G2 and G3 sequences in the GenBank database.

**Conclusion:**

The genotype of adult *E. granulosus* was similar to larval stage genotypes of parasite (G1-G3 complex).

## Introduction

Cystic Echinococcosis (CE) is one of the most common parasitic zoonosis caused by *Echinococcus granulosus* worldwide (1, 2). CE makes many economic problems in animal and human society and it constitutes major public health issues. A seropositivity rate in different parts of Iran country was within 1.2%-21.4% (3). The infection rate was reported in dogs from 5% to 49% in different regions of Iran (3).Within the life cycle of E*. granulosus*, canine and wild carnivores can serve as important definitive hosts.

Nowadays, there are 10 distinct genetic types (Genotypes G1-G10) of different *E. granulosus* (4, 5). The most isolate of human *E. granulosus* have been indicated to be common with sheep strain (6, 7). Epidemiological studies along with genetic characterization have demonstrated the prevalence of CE and some particular genotypes of *E. granulosus* in relation to type of livestock host. Knowing the genetic varieties of the parasite has some effects on epidemiology, pathology, infectivity, and control of hydatidosis (8). The susceptibility of human to CE may be correlated with *E. granulosus* genotype (5). Therefore, it could be very important to understand the levels of genetic variability and phylogenetic relationships among and within *E. granulosus* genotypes and this by itself has an important role in programs for infectious disease control, especially for health’s organization (9).

This study investigated the molecular genetic diversity of adult’s worm of *E. granulosus* by using NADH dehydrogenase gene subunit1 (*nad1*) among stray dogs and golden jackals in Ilam province West of Iran.

## Materials and Methods

### Parasite

**Fig. 1:**
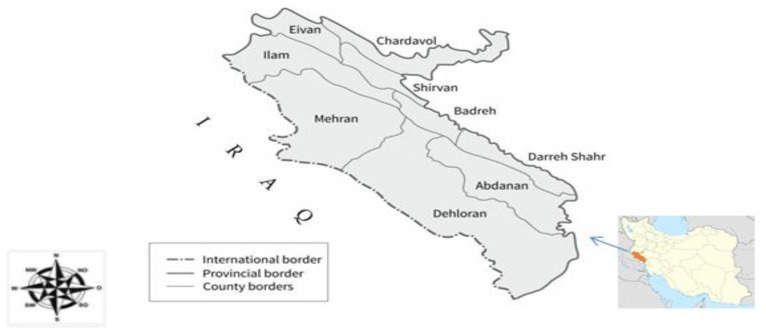
The collection sites of canine examined from Ilam Province between 2012 and 2014

From Feb 2012 to Oct 2014, 75 stray dogs, 73 golden jackals, and 70 red fox carcasses killed in car accidents were collected from the road in Ilam Province (Fig.1). After necropsy, the intestines of canine were evaluated for the presence of adult worms of *E. granulosus*. The separated worms from each infected animal were placed in a tube, rinsed three times with phosphate-buffered saline, and preserved in 80% ethanol until further analysis.

### Compliance with Ethical Standards:

This study was financially supported by the Tarbiat Modares University, Tehran, Iran. All procedures performed in studies involving animals were in accordance with the ethical standards of the Tarbiat Modares University Ethical Committee. All applicable international, national, and/or institutional guidelines for the care and use of animals were followed.

### DNA extraction

The study was done in Parasitology Department of Medical Sciences Faculty of Tarbiat Modares University, Tehran, Iran in 2012-14. Twenty-two samples (twenty samples from stray dogs and two from golden jackals) were examined for DNA extraction. The adult worms were washed three times using sterile distilled water to remove ethanol. Each sample contains 10 *E. granulosus* from each infected animals, randomly selected and provided for repeated freezing and thawing, as well as abrasion and mechanical grinder. Genomic DNA was extracted according to the manufacturer’s instruction by using DNA extraction kit (Geneall, Korea). Concentration of DNA was determined by spectrophotometry and the isolates were eluted in 50 μl of ddw at 70 °C and stored at −20 °C until PCR reactions.

### Molecular analysis

The isolates were analyzed using amplification of mtDNA*nad*1 gene. PCRs were carried out in a final volume of 25 μl including (10X buffer 2.5 μl, genomic DNA 4 μl, MgCl2, 1.75 μl, 0.6 μl of each deoxynucleotide triphosphate (dNTPs), 1 μl of each primer, Taq DNA polymerase 0.15 μl and distilled water 14 μl) (10).

The forward (nad1.F) JB11: 5′-AGATTCGTAAGGGGCCTAATA-3′and reverse (nad1.R) JB12: 5′-ACCACTAACTAATTCACTTTC-3′primers were employed for PCR amplification. The following temperature profile was used for DNA amplification. Initial denaturation 5 min for 94 °C, denaturation1minfor 94C; annealing 1minfor 50C; extension 1minfor 72 °C, number of cycles 35 and final extension 10 min for 72 °C.

PCR products were loaded on 1.2% (w/v) agarose gel (Sinnagen, Iran) in SB buffer (10 mM Sodium hydroxide, pH adjusted to 8.5 with Boric acid) (11) and stained with 0.5 μg/ml ethidium bromide. Electrophoresis was carried out for 50 min at 80 V. The bands were visualized in UV transilluminator and digitally were photographed (10, 12).

### PCR-RFLP and DNA Sequencing

This method was done on PCR products mtDNA*nad1* regions of isolates from animals with 3-base cutting restriction endonucleases, *Alu*I, *Rsa*I and *Hpa*II. Electrophoresed products were stained with 0.5μg/ml ethidium bromide and then the created bands were photographed and observed by the documentation gel system. In order to confirm RFLP results, a number of PCR Product were randomly bi-directionally sequenced using PCR primers by the Seqtech Company in the USA. Results were analyzed by Mega ver. 6 software package and compared with the recorded results in GenBank.

## Results

**Fig. 2:**
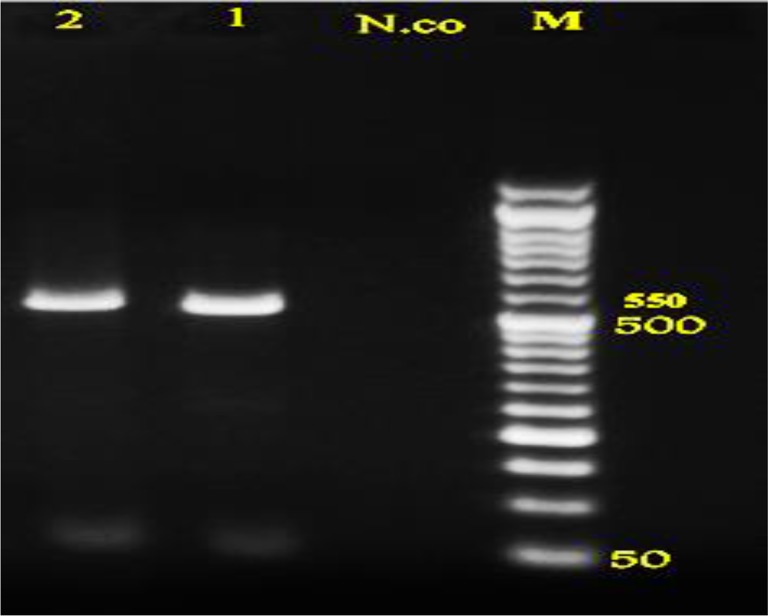
PCR products of mtDNA-*nad1* of adult *E. granulosus*on agar gel: M: marker with 50bp molecular weight, Co.n: control negative, 1: dog, 2: jackal samples

Totally, twenty stray dogs out of 75 (26.66%) and two out of 73 (2.74%) golden jackal showed infection with *E. granulosus*. No foxes revealed to be infected. *Nad1* gene was successfully amplified for all isolates. Amplified fragment size of *nad1* gene of dogs and jackals isolates both showed approximately 550bp amplicons in PCR reaction (Fig.2).

**Fig. 3:**
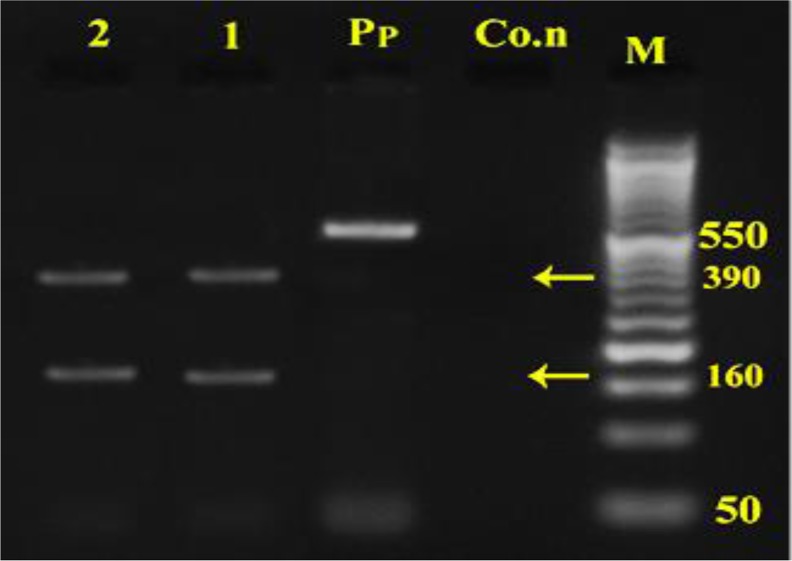
Digestion pattern of 550bp PCR products of mtDNA*nad-*1 fragment with *Alu*I enzyme on agar gel M: marker with 50bp molecular weight, Co.n: negative control, Pp: PCR products without enzyme, 1: dog, 2: jackal samples

**Fig. 4:**
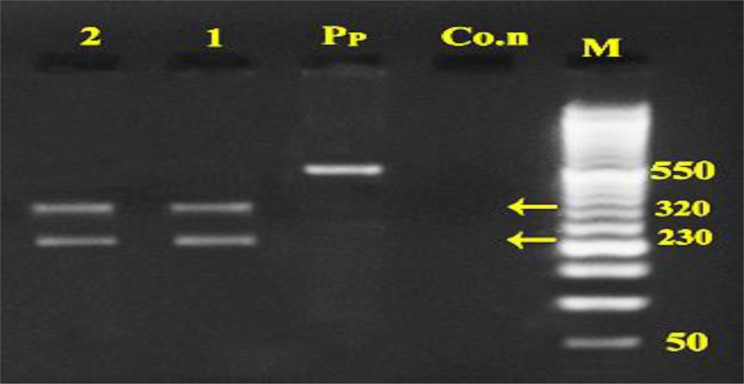
Digestion pattern of 550bp PCR products of mtDNA-*nad1* fragment with *Rsa*1 enzyme on agar gel M: marker with 50bp molecular weight, Co.n: negative control, Pp: PCR products without enzyme, 1: dog, 2: jackal samples

**Fig. 5:**
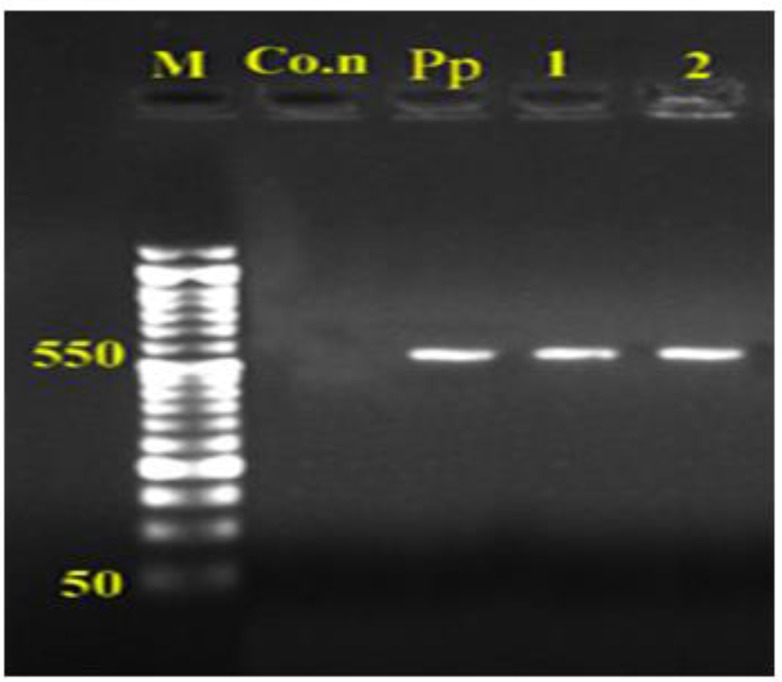
Digestion pattern of 550bp PCR products of mtDNA-*nad1* fragment with *Hpa*II enzyme on agar gel M: marker with 50bp molecular weight, Co.n: negative control, Pp: PCR products without enzyme, 1: dog, 2: jackal samples

**Fig. 6:**
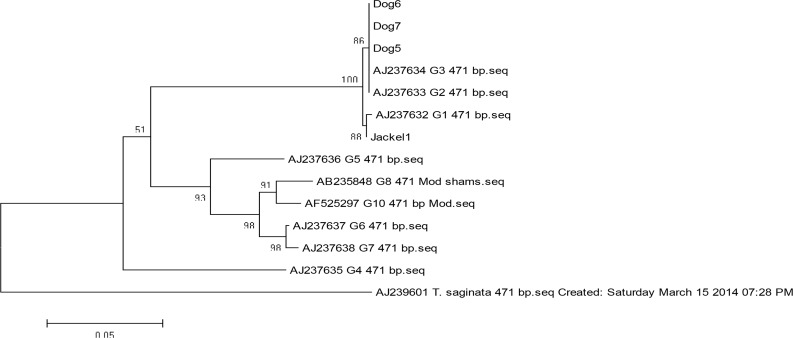
Phylogenetic relationships among *E. granulosus* isolated from dogs and jackals based on mtDNA *nad1* gene sequence. The evolutionary history was inferred using the neighbor-joining method, supported by 1000 bootstrap replicates

The *Alu*1 digested the product into two bands for both dog and jackal isolates of approximately 160bp and 390bp fragments (Fig.3) while the *Rsa*1 cut the product into 320bp and 230bp fragments for both dog and jackal samples (Fig.4). The *Hpa*II restriction enzyme had no effect on the PCR product for *nad*1 and after digestion intact 550bp fragment was seen (Fig.5). Sequence data of amplified fragment (471bp) of ten isolates from dogs and two isolates from jackal showed 100% homology with GenBank reference sequence for G1 (AJ237632), G2 (AJ237633) andG3 (AJ237634) genotypes (Fig.6). Phylogenic tree was drawn and the similarity of jackal samples with G1genotype (sheep strain), dog samples with G2 genotype (Tasmania sheep strain) and G3 genotype (Buffalo strain) (Fig.6). Therefore, the presence of G1–G3 complex of *E. granulosus* was confirmed in Ilam Province. The sequence data were registered into GenBank reference sequences with Accession Nos. (KT338943 for dog isolates and KT338944 for jackal samples).

## Discussion

Iran has geographically been located in high prevalence region of echinococcosis. The infection rate of hydatidosis in cattle, sheep, and goat based on abattoir data in Western Iran (Ilam, Lorestan, Kermanshah and West Azerbaijan provinces), were 11.1%, 16.4%, 12.4% and 6.3%, respectively (13). The rate of infection in stray dogs was reported 22.3% in the north of Iran, 17.6% in Kerman, 22% in Mashhad, 12.5 % in the west Azerbaijan, 11.4% in Kordestan, 16.7% in Kermanshah, 30.9% in Lorestan and 9% in Ilam (14). However, spillover events from domestic to sylvatic cycles are suspected to be frequent in areas where infected viscera cheered livestock are known to be accessible to jackals and foxes (13).

Up to date, some studies have been performed on the molecular characterization of *E. granulosus* isolates from humans, sheep, goats, cattle, and camels in different parts of Iran (15-40). The result of this type of studies indicated the presence of G1, G2, G3 and G6 genes in our country (21-40). In a study that conducted in Ilam Province, three genotypes were detected as G1, G2, and G3 (17). In this study, on mtDNA of *nad1*gene of adult worms, the results were similar to that of others. G1 genotype has been introduced as a dominant variant in Iran by some studies (18, 19, 21, 22, 24, 27). In addition, the presence of camel (G6 genotype) strains has been reported in Iran (16, 18, 20, 30, 31). Sequencing of the *nad1* and *cox1* genes and *ITS1*-PCR coupled to RFLP confirmed these observations. The same geno-type for sheep and human isolates, while different ones for camel (18). The genotype diversity of three mitochondrial genes *cox1*, *nad1* and *atp6* was investigated and also partial sequences of 12S rRNA gene in their isolates and confirmed the presence of G1 and G6 genotypes in different intermediate hosts, including cattle, camels, sheep, buffalo and goats in different geographic areas in Iran (21). Among 55 samples from domestic animals and humans in Ardabil, the G1 was the dominant strain in Iran and G3 genotype was reported in two human isolates (22).

The present study indicated the presence of (G1-G3 complex) of *E. granulosus* among dogs and jackals in Ilam Province. In a molecular study, all human and sheep were reported to be of (G1-G3 complex) genotypes (17). By analyzing *cox1* and *nad1* gene sequences of the adult worms isolated from dogs in Lorestan near to Ilam, about 75% of the cases specified as G1, 10% as G2 and only 15% as G3 geno-types (23). These findings are, more or less, in accordance with our research results. Our findings also were confirmed by two studies that all human isolates was reported as G1 genotype (24, 25). G1, G2 and G3 are the most common genotypes of the parasite in intermediate hosts such as sheep, camel, buffalo and occasionally human throughout the world (1, 6, 7, 21, 22). In addition to stray dogs and golden jackals were found to be infected with G1, G2, and G3 genotypes. By this study, G1 genotype in golden jackal has been reported for the first time in the world.

## Conclusion

The infected definitive hosts (dogs and jackal) were harbored the G1-G3 genotypes are predominant in the transmission cycle of E*. granulosus* in West of Iran.
